# Application of a Lifestyle-Based Score to Predict Cardiovascular Risk in African Americans: The Jackson Heart Study

**DOI:** 10.3390/jcm10112252

**Published:** 2021-05-22

**Authors:** Mercedes Sotos-Prieto, Songzhu Zhao, David Kline, Guy Brock, Holly Gooding, Josiemer Mattei, Fernando Rodriguez-Artalejo, Yuan-I Min, Eric B. Rimm, Katherine L. Tucker, Joshua J. Joseph

**Affiliations:** 1 Department of Preventive Medicine and Public Health, School of Medicine, Universidad Autónoma de Madrid, IdiPaz (Instituto de Investigación Sanitaria Hospital Universitario La Paz), Paseo de la Castellana, 261, 28046 Madrid, Spain; fernando.artalejo@uam.es; 2 CIBERESP (CIBER of Epidemiology and Public Health), Av. Monforte de Lemos, 3-5, 28029 Madrid, Spain; 3 Department of Environmental Health, Harvard T.H. Chan School of Public Health, 665 Huntington Avenue, Boston, MA 02115, USA; 4 IMDEA-Food Institute, CEI UAM + CSIC, Crta. de Cantoblanco 8, E, 28049 Madrid, Spain; 5 Department of Biomedical Informatics and Center for Biostatistics, The Ohio State University, 320 Lincoln Tower, 1800 Cannon Drive, Columbus, OH 43210, USA; songzhu.zhao@osumc.edu (S.Z.); david.kline@osumc.edu (D.K.); guy.brock@osumc.edu (G.B.); 6 Department of Pediatrics, Emory University School of Medicine, 100 Woodruff Circle, Atlanta, GA 30322, USA; holly.gooding@emory.edu; 7 Department of Nutrition, Harvard T.H. Chan School of Public Health, 665 Huntington Avenue, Boston, MA 02115, USA; jmattei@hsph.harvard.edu; 8 Department of Medicine, University of Mississippi Medical Center, 2500 North State Street, Jackson, MS 39216, USA; ymin@umc.edu; 9 Department of Epidemiology, Harvard T.H. Chan School of Public Health, 665 Huntington Avenue, Boston, MA 02115, USA; erimm@hsph.harvard.edu; 10 Channing Division of Network Medicine, Brigham and Women’s Hospital and Harvard Medical School, 75 Francis Street, Boston, MA 02115, USA; 11 Department of Biomedical and Nutritional Sciences, University of Massachusetts Lowell, 3 Solomont Way, Suite 4, Lowell, MA 01854, USA; katherine_tucker@uml.edu; 12 Division of Endocrinology, Diabetes and Metabolism, The Ohio State University Wexner Medical Center, 579 McCampbell Hall, 1581 Dodd Drive, Columbus, OH 43210, USA; joseph.117@osu.edu

**Keywords:** lifestyle, risk assessment, prevention, African Americans, Blacks

## Abstract

Cardiovascular disease (CVD) primordial prevention tools applicable to diverse populations are scarce. Our aim was to assess the performance of a lifestyle-based tool to estimate CVD risk in an African American population. The Jackson Heart Study is a prospective cohort including 5306 African American participants in Jackson, Mississippi (2000–2004), with a mean follow up of 12 years. The Healthy Heart Score is a lifestyle-based CVD risk prediction model based on nine components: body mass index (BMI), physical activity, smoking, and a 5-component diet score. Gender-specific beta coefficients from its derivation cohorts were used to assess the performance of the Healthy Heart Score. Model discrimination was assessed using Harrell’s C-Index for survival data and time dependent Area Under the Curve. Model calibration was evaluated through calibration plots. A total of 189 CVD events occurred. The Healthy Heart Score showed high-moderate discrimination for CVD events (C-statistic 0.75 [95% CI, 0.71–0.78]) but with little improvement over the age-only model. Both the age-only and Healthy Heart Score models had better performance in participants without diabetes at baseline and showed good calibration. In African Americans, the Healthy Heart Score does not improve prediction of mid-life CVD events beyond what is obtained by age alone.

## 1. Introduction

African Americans have a disproportionally higher risk of cardiovascular disease (CVD) compared to non-Hispanic white adults [1,2,3]. Clinical risk factors, such as diabetes mellitus, hypertension, and dyslipidemia, which are associated with substantial long-term risk of CVD, are often elevated in African Americans [2,4]. Additionally, African American are less likely than other racial/ethnic groups to achieve ideal cardiovascular health which includes also healthy diet, physical activity, or healthy weight [2,4].

Existing CVD risk prediction tools are critically debated regarding their applicability to African Americans because most have been developed in non-Hispanic white cohorts. More recently, Fox et al. [5] developed and assessed several risk estimation algorithms for all CVD in more than 5000 African Americans from the Jackson Heart Study (JHS) cohort, and concluded that the current Framingham Risk Score [6,7] and the pooled cohort risk algorithms [8] worked well in Blacks so that a distinct calculator might not be necessary. However, these prediction tools [5,9,10] include clinical risk factors such as blood lipids or blood pressure to estimate an individual’s ten-year risk of CVD, which often underestimate risk burden in young adults (<50 years) and in the longer term. It is known that modifiable lifestyle factors are major determinants of CVD and other chronic diseases, and that African Americans report unhealthier dietary behaviors and poorer cardiovascular health metrics as defined by the American Heart Association (AHA) than the general U.S. population [1,3,4,5,6,7,8,9,10,11]. These important health disparities are reflective of longstanding discriminatory and racist policies in the US [12].

Given the importance of reducing CVD risk in this population, a focus on lifestyle factors such as diet, physical activity, smoking, and weight status is key to preventing the emergence of clinical risk factors (primordial prevention) and CVD risk in the long term. The Healthy Heart Score is a 20-year CVD risk prediction tool based on nine modifiable health behaviors that has been derived in mostly non-Hispanic white adults (mean age 52 years) and has shown good discrimination (C statistic, 0.72 in men and 0.77 in women) [13]. More recently, Gooding et al. observed moderately good performance when applying the Healthy Heart Score to younger persons (mean age 24.8 years) without CVD (C statistic 0.71); the model performed better in white than Black individuals (C statistic 0.77 vs. 0.64) [14]. However, the Healthy Heart Score has not yet been validated in an African American population. Therefore, using data from the JHS cohort, we aimed to assess the performance of the Healthy Heart Score among African Americans to estimate the risk of CVD events.

## 2. Materials and Methods

### 2.1. Study Design and Participants

The JHS is a longitudinal cohort study of CVD in African Americans. Between September 2000 and March 2004, 5306 African American adults, 21 years and older, were recruited in the tri-county area of Jackson, Mississippi. Details about the study design and data collection were published elsewhere [15]. Briefly, participants were selected to include people representative of the African American population in the Jackson area, in terms of age, sex, and socioeconomic status. At baseline, data were collected on demographic characteristics, socioeconomic status, behavioral factors, morbidity, history of myocardial infarction (MI) and stroke, parental history of hypertension, and cardiac testing. During the clinic visit, an inventory of prescription and over-the-counter medications taken within two weeks before the study examination was conducted; height, weight, and blood pressure (BP) were measured; and blood and urine specimens were collected. For the present analysis, participants were included if they had available data (i.e., information about the main components of the Healthy Heart Score) to calculate the Healthy Heart Score (*n* = 4665). Participants with CVD were excluded (*n* = 1493) and those lost to follow-up (*n* = 131). The final sample size for the current analysis was 3041.

### 2.2. Healthy Heart Score

The original Healthy Heart Score was developed for predicting 20-year risk of CVD and derived within two cohorts that included mostly non-Hispanic white adults (61,025 women in the Nurses’ Health Study (NHS) and 34,478 men in the Health Professionals Follow-up Study (HPFS)) free of CVD, diabetes, and cancer at baseline [13]. Although the initial model included various dietary and lifestyle factors, the most parsimonious, rather than comprehensive, model for predicting 20-year risk of CVD was selected. This model, in addition to age (years), included the following lifestyle factors: smoking status (current smokers or past smokers), body mass index (BMI, kg/m^2^), physical activity (moderate/vigorous hours/week), and intake of cereal fiber (grams/day), fruit and vegetables (servings/ day), red and processed meat (servings/day), nuts (servings/day), sugar-sweetened beverages (serving/day), and alcohol consumption (grams of alcohol) (Figure S1). The final Healthy Heart Score was calculated, and a higher score indicated higher risk of CVD.

The Healthy Heart Score was derived and calculated separately, by sex, in the JHS cohort, using validated questionnaires and the specific beta coefficients from the original derivation cohort (Figure S1). Diet components were derived using a 158-item food frequency questionnaire specifically developed for this region, and validated against 24 h recalls and biomarkers in this population [16,17,18]. Physical activity was estimated through previously published methods [15,19]. Specifically, the physical activity questionnaire assessed time spent in moderate and vigorous activity during the previous seven days, and assessed four different domains of activity (active living, work, home and garden, and sport and exercise). Briefly, hours per week of moderate to vigorous activity was calculated by considering the metabolic equivalent tasks (MET) for each activity. Moderate activity was defined as 3 to 6 MET and vigorous activity as >6 MET. BMI was calculated from weight (kg) divided by the square of height (meters). Smoking status, age, sex, and race were self-reported via questionnaire [15].

### 2.3. Assessment of Cardiovascular Disease

For the present study, we defined CVD incidence as the first occurrence between the date of a participant’s first visit and 31 December 2016 of any of following four outcomes: fatal/non-fatal myocardial infarction (MI), stroke, or fatal coronary heart disease (CHD) [20]. Incident MI, fatal CHD, and stroke were determined through adjudication on review of relevant medical records. The annual follow-up included interviews with participants and next of kin to ascertain health events, such as cardiac events, and hospitalizations or death through questionnaires completed by physicians, medical examiners, or coroners, and reviewed by the medical record abstraction unit to generate diagnostic information. These diagnoses were reviewed and adjudicated by trained medical personnel. CVD hospitalizations were identified and adjudicated as described elsewhere [21]. Death certificates from state vital statistics offices were assessed for potential CVD events. The self-reported data from the annual follow-up were reconciled with the hospital discharge index data. The primary diagnoses based on International Classification of Diseases-9th Revision-Clinical Modification codes were reviewed and adjudicated by trained medical personnel. We tracked the occurrence of CHD and stroke between 2000 and 2016.

### 2.4. Statistical Analysis

Participant characteristics were summarized using descriptive statistics including the mean (standard deviation, SD) or median (interquartile range) for continuous variables, and frequencies for categorical variables. Cox proportional hazards models were used to assess time to cardiovascular event, and hazard ratios (HRs) and 95% confidence intervals (CIs) were calculated. The external validation of the Healthy Heart Score in the JHS was based on the discrimination and calibration of the model using the sex-specific beta-coefficients and baseline hazards derived from the NHS and HPFS. Model discrimination was assessed using Harrell’s C-statistic for survival data, where larger values indicate better discrimination [22], while time-dependent area under the curve (AUC) analyses were used to evaluate the discriminative performance of models over time. We compared the discrimination of the Healthy Heart Score first with an age-only model, and then with the separate components of the score included in a multivariable model. In sensitivity analysis, we also assessed model performance stratifying by median age at baseline and stratifying by baseline diabetes status. Finally, model calibration was assessed using plots of the average predicted risk within deciles of predicted risk against the observed risk in that decile.

Statistical significance was set at two-sided *p* value <0.05. All analyses were performed using SAS software (version 9.4 SAS Institute INC, Cary, NC, USA).

## 3. Results

The analytic sample consisted of 3041 participants, of which 53.5% were women and had a mean (SD) age of 53.1 (12.7) years. Baseline characteristics of participants in the JHS, overall and separately by sex, are shown in Table 1. The diet score mean was −2.5 (4.5), less favorable than in the NHS and HPFS but similar to the CARDIA study (Table S1). The mean (SD) of the Healthy Heart Score was 7.7 (1.3), similar to the NHS/HPFS (Table S1). BMI was higher, compared to the original cohort from which the Healthy Heart Score was developed 31.7 (7.3) vs. 25.1 (4.5) kg/m^2^ in women, and 29.4 (5.9) vs. 25.4 (2.9) kg/m^2^ in men. The JHS also reported lower physical activity, as well as higher consumption of sugar-sweetened beverages and red and processed meats (Table S1).

**Figure 1:**
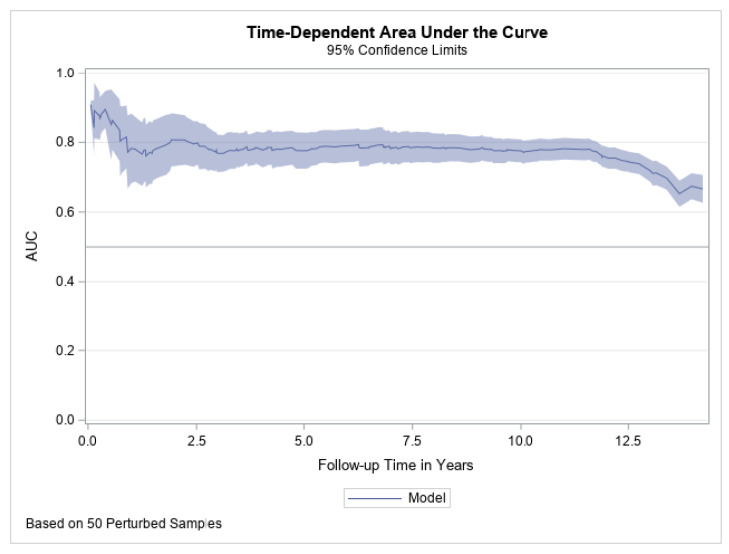
AUC Plot with 95% Confidence Limits to examine model performance of the Healthy Heart Score for Cardiovascular Events over 12 years.

During a mean follow-up of 12 years, there were 189 incident cases of CVD (111 in women and 78 in men, 110 CHD, 79 stroke). Model performance of the Healthy Heart Score for the overall participants and by sex are presented in Table 2. The C-statistic was 0.74 for both the CVD risk model including age only, and the Healthy Heart Score, and slightly better for women compared to men (C-statistic 0.75 vs. 0.73, respectively). The Healthy Heart Score seemed to be somewhat more discriminative within the first year of follow-up and remained fairly constant in subsequent years (Figure 1). The time dependent AUC analysis showed similar results, with better discrimination in the first year and with the complete model (age only: AUC 0.76 [95% CI, 0.65–0.88] vs. Healthy Heart Score 0.78 [95% CI 0.68–0.89]).

The C-statistic and its 95% CI for the multivariate model including each of the Healthy Heart Score components separately are shown in Table S2. In multivariable analyses, a 1-unit increase in diet score was associated with a 5% decrease in CVD in men and 2% decrease in women (*p* < 0.005) (Table S2). Based on the results, fitting a model to the full cohort and re-estimating the parameters showed little gain from the Healthy Heart Score, so cohort-specific coefficients were not needed.

We assessed the performance of the Healthy Heart Score stratifying by median age (<52 vs. ≥52 years). In contrast to the model with age only, the Healthy Heart Score C-statistic increased from 0.61 (95% 0.53–0.68) to 0.67 (95% CI, 0.59–0.75) among the younger participants (Table 3). We further compared model discrimination between those with and without diabetes at baseline (Table S3). Both the age-only model and Healthy Heart Score had better model performance in participants without diabetes.

**Figure 2:**
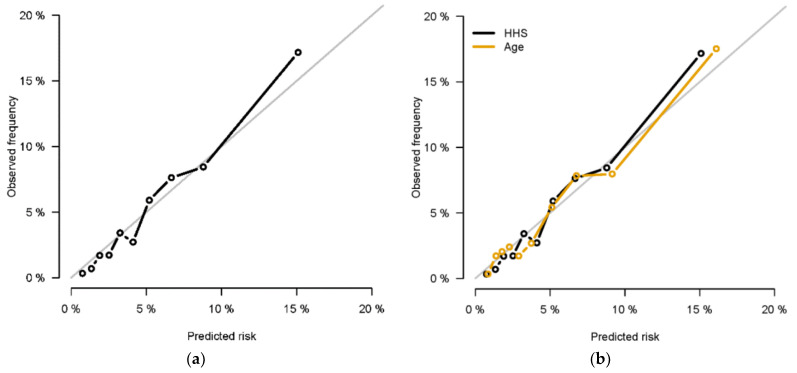
(**a**) Calibration Plot of Observed to Estimated risk of CVD for model with the Healthy Heart Score (HHS). (**b**) Calibration Plot of Observed to Estimated risk of CVD comparing HHS model vs. age alone model (plotting the average predicted risk within deciles of predicted risk observed risk in that decile). The diagonal line represents a perfect calibration.

The model with the Healthy Heart Score had good calibration (Figure 2; all points were close to the diagonal line, which represents a perfect calibration).

## 4. Discussion

In this African American population, the Healthy Heart Score, a lifestyle-based CVD risk prediction tool performed moderately well to estimate CVD events (C-statistic 0.77) and performed better among those without versus with diabetes at baseline (C-statistic 0.75 vs. 0.62). However, the age-based model barely changed when all the separate components of the Healthy Heart Score were included. The JHS is a population that has a higher prevalence of comorbidities than the original cohort where the Healthy Heart Score was developed. For example, in the NHS/HPFS [13], around 20% of the participants had hypertension or hypercholesterolemia while in the JHS, the prevalence of hypertension was close to 50%, and BMI was considerably higher (JHS: ~31 kg/m^2^ vs. NHS/HPFS: ~25 kg/m^2^). The fact that the NHS and HPFS the group with clinical risk factors showed lower C-statistics suggests that the Healthy Heart Score works better in participants without clinical risk factors. This may be why, in our cohort, performance with the complete model did not add much to the age-only model due to the high prevalence of morbidities. Further, the predictive value of lifestyle factors may be diminished after the development of clinical risk factors. Indeed, our results showed a higher C-statistic and AUC in those participants without diabetes. This also corresponds with the CARDIA study where, in younger persons without clinical risk factors, the Healthy Heart Score performed moderately well [14]. Similarly, in line with our results, the addition of the Healthy Heart Score components to age increased the C-statistic from 0.67 to 0.77 in non-Hispanic whites, but improved it only slightly (from 0.63 to 0.66) in African Americans. Of note, CARDIA included younger participants with fewer morbidities. We also stratified by age, and discrimination was greater in younger versus older ages, as well as early in the follow-up.

Cox models showed that diet and smoking, but no other components of the score, were significantly associated with risk of CVD. Previous studies in the JHS evaluating Life’s Simple 7, a cardiovascular health metric that includes both clinical (blood pressure, total cholesterol, fasting glucose) and lifestyle factors (smoking, physical activity, and BMI), showed that participants with higher cardiovascular health had lower risk of hypertension [20] and risk of diabetes [23,24]. However, fewer African Americans than whites maintained four or more optimal health behaviors until age 50 years, and maintenance thereafter was low [25]. In addition, another study compared the contribution of changes in modifiable risk factors versus aging to the development of high ten-year predicted CVD risk in African Americans and found that increases in systolic blood pressure and antihypertensive medication initiation were the major contributors, especially in those <50 years, and that their contributions were similar to that of age [26]. In our population, almost half of the participants had hypertension, which is known to increase the risk of CVD events [27]. Therefore, preventing the development of those biological risk factors in the first place is paramount for African Americans, who exhibit disproportionately higher rates of CVD compared to other racial/ethnic groups [2]. The Healthy Heart Score was created to be a primordial prevention tool, to help prevent the initiation of risk factors. Previous studies with the Healthy Heart Score showed that women in the highest quintile (worst behaviors) of the Healthy Heart Score had an estimated 18 times higher risk of diabetes, 5 times of hypertension, 3 times of hypercholesterolemia, and 53 times of all three risk factors combined, compared to women in the lowest quintile [28], as well as higher all-cause mortality [29].

The extant literature suggests that the role of lifestyle in the prevention of CVD or its risk factors may differ between races. For example, in the Women’s Health Initiative [30], a healthier diet pattern, quantified by the Alternate Healthy Eating Index, was associated with lower risk of diabetes among non-Hispanic whites and Hispanic Americans, but not African Americans or Asian Americans; also, the Dietary Approaches to Stop Hypertension (DASH) diet lowered blood pressure more effectively among African Americans than non-Hispanic whites in a clinical trial [31]. In the ARIC study, alcohol intake was inversely associated with CHD risk in white men and women, but positively associated with risk in African American men [32]. In our study, the Healthy Heart Score included a composite diet score of only five items along with other lifestyles. Whether other lifestyle factors not captured in this tool might be more relevant in this population, such as social determinants of health, racism, discrimination, or environmental influences, remain to be elucidated.

Limitations of the study include the self-reported nature of the lifestyle variables that could lead to misclassification; however, questionnaires showed good validation [33,34]. The Healthy Heart Score is meant to be a primordial prevention tool, therefore excluding participants at baseline with any clinical risk factor that could have improved the performance; however, due to the high prevalence of hypertension (45.1%) and hypercholesterolemia (19%) in this population, excluding them would have led to a small number of CVD cases and low statistical power. However, we did test performance by diabetes status, and the performance was better among those without diabetes (C-statistic 0.61 vs. 0.77 for those with diabetes or without, respectively), underscoring the importance of using this tool before the clinical risk factors develop.

Although other behavioral factors may be important in the development of CVD and mortality in this population, we did not examine their potential role because our aim was to assess the Healthy Heart Score, and it was outside the scope of this study. The duration of the follow-up of the JHS limits the ability to ascertain long-term CVD events (20 years or more); however, we used one of the most comprehensive cohorts of African Americans with a relatively high rate of CVD incidence (CARDIA 98 cases in blacks vs. 189 in the JHS). In addition, not only do the JHS population characteristics differ from the NHS/HPFS or CARDIA, but the latter cohorts were started in the mid-1980s, while JHS is more contemporary (recruitment was in 2000–2004). Changes in clinical risk factors (diabetes, hypertension, obesity), especially in the Southern U.S. over the follow-up period of the JHS, have been reported, which may influence the ability of the Healthy Heart Score calculated at baseline to predict CVD incidence [35].

In conclusion, the Healthy Heart Score performance did not show substantial improvement beyond the age-based-only model in predicting CVD risk; this may be due to the high degree of early hypertension, hypercholesterolemia, and diabetes already existing in this population at baseline. Further longitudinal studies in African Americans without clinical risk factors at baseline, and including other social determinants of health and discrimination known to disproportionately affect African American people, are required.

## Figures and Tables

**Table 1:** Baseline demographics of participants in the Jackson Heart Study, by sex and overall *.

Variable	Total (*n* = 3041)	Female (*n* = 1917)	Male (*n* = 1124)
Age, years, mean (SD)	53.1 (12.7)	53.5 (12.8)	52.4 (12.5)
Smoker			
Never	2189 (72%)	1491 (77.8%)	698 (62.1%)
Past	492 (16.2%)	253 (13.2%)	239 (21.3%)
Current	360 (11.8%)	173 (9%)	187 (16.6%)
Body mass index, kg/m^2^	30.9 (6.9)	31.9 (7.3)	29.4 (5.9)
Moderate and vigorous physical activity, hours/week	0.3 [0, 1.7]	0.2 [0, 1.5]	0.5 [0, 1.9]
Total alcohol intake, g/day	0.4 [0.1, 1.8]	0.3 [0.1, 0.9]	0.9 [0.1, 7.4]
Total Cholesterol, (mg/dL) mean (SD)	199.9 (38.1)	200.8 (37.6)	198.5 (38.9)
Low-Density Lipoprotein (mg/dL) mean (SD)	128.2 (35.8)	126.6 (34.9)	131 (37)
Estimated Glomerular Filtration Rate, ml/min per 1.73 m^2^ mean (SD)	96.6 (19.4)	97.5 (20.4)	95.2 (17.6)
Blood pressure, mmHg mean (SD)	125.7 (16.4)	125 (16.8)	127 (15.5)
Glucose, mg/dl mean (SD)	90.1 (8.7)	89.3 (8.7)	91.4 (8.5)
Hemoglobin A1c, %	5.5 [5.2, 5.8]	5.5 [5.2, 5.8]	5.6 [5.2, 5.8]
*Diet Score Components*			
Total fruits and vegetables, serving/day	5.9 [3.8, 8.8]	6 [3.9, 9]	5.7 [3.7, 8.5]
Total cereal fiber, g/day	1.1 [0.4, 2.8]	1.2 [0.4, 2.9]	1.1 [0.4, 2.6]
Total red and processed meat, serving/day	1.6 [0.9, 2.8]	1.4 [0.8, 2.4]	2.1 [1.2, 3.5]
Total nuts, serving/day	0.2 [0, 0.6]	0.1 [0, 0.5]	0.2 [0.1, 0.6]
Sweetened beverages, serving/day	1 [0.3, 2.2]	0.9 [0.3, 2]	1.1 [0.5, 2.5]
Diet Score, [score range]	−2.5 [−34.9, 15.2]	−1.9 [−34.9, 15.2]	−3.4 [−27.8, 4.3]
*Heart Healthy Score*			
Heart Healthy Score, [score range]	7.7 [3.4, 13]	7.4 [3.4, 13]	8.1 [4.4, 12.4]
*Events*			
Cardiovascular Events			
No	2852 (93.8%)	1806 (94.2%)	1046 (93.1%)
Yes	189 (6.2%)	111 (5.8%)	78 (6.9%)

* Median (Interquartile Range) or percentages are listed; *p*-values were calculated using Chi-Square (categorical variables), ANOVA (for normally distributed continuous variables), and Kruskal-Wallis test (for non-normally distributed continuous variables).

**Table 2:** Model performance of the Healthy Heart Score in the JHS for Cardiovascular Events in the overall cohort, men and women.

Incident Cardiovascular Events	Total (*n* = 3041)	Men (*n* = 1124)	Women (*n* = 1917)
HR (95% CI) Age Alone	1.07 (1.06, 1.09)	1.07 (1.05, 1.09)	1.08 (1.06, 1.1)
HR (95% CI) Healthy Heart Score	1.98 (1.77, 2.23)	2.07 (1.68, 2.55)	1.95 (1.7, 2.25)
Harrell’s Concordance Statistic (95% CI)—Age Alone	0.74 (0.7, 0.77)	0.73 (0.68, 0.79)	0.75 (0.7, 0.79)
Harrell’s Concordance Statistic—(95% CI) Healthy Heart Score	0.75 (0.71, 0.78)	0.73 (0.68, 0.78)	0.75 (0.71, 0.8)
Time Dependent AUC (95% CI)—Age Alone			
1 year	0.76 (0.65, 0.88)	0.81 (0.67, 0.95)	0.72 (0.54, 0.9)
3 years	0.76 (0.70, 0.83)	0.76 (0.65, 0.87)	0.77 (0.69, 0.84)
5 years	0.77 (0.72, 0.81)	0.76 (0.67, 0.84)	0.78 (0.72, 0.83)
Integrated Time-Dependent AUC—Age Alone	0.76	0.77	0.76
Time Dependent AUC (95% CI)—Healthy Heart Score			
1 year	0.78 (0.68, 0.89)	0.79 (0.64, 0.94)	0.76 (0.59, 0.93)
3 years	0.77 (0.72, 0.83)	0.78 (0.68, 0.87)	0.78 (0.7, 0.85)
5 years	0.78 (0.73, 0.82)	0.77 (0.69, 0.85)	0.78 (0.72, 0.84)
Integrated Time-Dependent AUC—Healthy Heart Score	0.77	0.77	0.77

**Table 3:** Model performance of the Healthy Heart Score in the Jackson Heart Study for Cardiovascular Events according to median of age for the overall cohort, men and women.

Incident Cardiovascular Events	Total (*n* = 3041)		Men (*n* = 1124)		Women (*n* = 1917)	
	Age < Median (*n* = 1520)	Age > = Median (*n* = 1521)	Age < Median (*n* = 562)	Age > = Median (*n* = 562)	Age < Median (*n* = 958)	Age > = Median (*n* = 959)
HR (95% CI) Age alone	1.08 (1.01, 1.14)	1.07 (1.05, 1.10)	1.08 (0.99, 1.17)	1.08 (1.05, 1.11)	1.09 (0.999, 1.18)	1.07 (1.05, 1.1)
Harrell’s Concordance Statistic—Age alone	0.61 (0.53, 0.68)	0.66 (0.62, 0.71)	0.61 (0.5, 0.73)	0.68 (0.61, 0.75)	0.62 (0.52, 0.71)	0.66 (0.60, 0.72)
Integrated Time-Dependent AUC—Age alone	0.64	0.68	0.69	0.70	0.59	0.66
HR (95% CI) Healthy Heart Score	1.6(1.24, 2.05)	1.97 (1.64, 2.38)	1.8 (1.08, 3)	1.87 (1.38, 2.54)	1.54 (1.05, 2.26)	1.94 (1.55, 2.44)
Harrell’s Concordance Statistic Healthy Heart Score	0.67 (0.59, 0.75)	0.67 (0.63, 0.71)	0.65 (0.54, 0.76)	0.66 (0.6, 0.73)	0.65 (0.55, 0.75)	0.67 (0.61, 0.73)
Integrated Time-Dependent AUC Healthy Heart Score	0.69	0.69	0.69	0.71	0.63	0.67

## Data Availability

Data are not publicly available and are available upon request: https://www.jacksonheartstudy.org/Research/Study-Data/Data-Access (accessed on 1 March 2020).

## Supplementary Materials

The following are available online at https://www.mdpi.com/article/10.3390/jcm10112252/s1, Figure S1: Formula to estimate the 20-Year Risk of CVD based on lifestyle predictors in women (Nurses’ Health Study) and men (Health Professionals Follow-up), Table S1: Comparison of the Healthy Heart Score components between participants in the JHS, NHS, HPFS, and CARDIA studies, Table S2: Model performance of Multivariate analysis including Lifestyle Factors, Table S3: Model performance of the Healthy Heart Score in the Jackson Heart Study for Cardiovascular Events or death for the overall in Participants with and without diabetes based on a) age only model; b) Healthy Heart Score.
