# Supplementary material for: Application of a Lifestyle-Based Score to Predict Cardiovascular Risk in African Americans: The Jackson Heart Study
Source: J Clin Med. 2021 May 22;10(11):2252. doi: 10.3390/jcm10112252 (PMC8196953; doi:10.3390/jcm10112252)
Supplement: Supplementary file 1 [file jcm-10-02252-s001.zip › jcm-1193361-Supplemental Material.pdf]

SUPPLEMENTAL MATERIAL

Table S1 - Comparison of the Healthy Heart Score components between participants in the JHS, NHS, HPFS and CARDIA studies[a]

	Men			Women		
	JHS	HPFS	CARDIA	JHS	NHS	CARDIA
Age, years	52.4 (12.5)	52 (9.0)	24.9 (3.6)	53.5 (12.8)	52.0 (7.0)	24.9 (3.7)
HHS components						
Smoking (%)						
Never	62	49	56.7	78	44	56.8
Past	21	42	12.7	13	35	14.3
Current	17	9	30.6	9	21	28.9
Body mass index, kg/m^2	29.4 (5.9)	25.4 (2.9)	24.4 (3.9)	31.9 (7.3)	25.1 (4.5)	24.6 (5.8)
Physical activity, h/wk	1.4 (2)	2.2 (2.1)	2.9 (2.5)	1.1 (1.9)	1.6 (2.0)	1.8 (1.9)
Alcohol, g/d	8 (17.8)	11.1 (14.5)	13.2 (17.5)	2 (7.1)	6.1 (9.9)	5.3 (9.5)
Dietary components						
Fruit and vegetables, serving/d	6.6 (4.1)	4.8 (2.6)	2.8 (2.3)	7.1 (4.6)	5.8 (2.6)	4.0 (3.1)
Sugar-sweetened beverages, serving/d	1.9 (2.3)	0.4 (0.6)	1.8 (2.2)	1.6 (2.1)	0.2 (0.5)	1.4 (2.0)
Red and processed meats, serving/d	2.7 (2.3)	1.0 (0.7)	2.8 (1.6)	1.8 (1.6)	0.9 (0.5)	2.5 (2.2)
Cereal fiber g/d	2 (2.6)	5.8 (3.2)	3.2 (3.4)	2.1 (2.6)	4.3 (2.4)	3.0 (3.0)
Nuts, serving/d	0.5 (0.7)	0.5 (0.6)	0.5 (0.7)	0.4 (0.7)	0.3 (0.4)	0.5 (0.8)
Total diet score	-3.4 (4.2)	1.0 (1.7)	-3.7 (4.0)	-1.9 (4.6)	3.6 (1.8)	-2.9 (5.5)
Total HHS[b]	8.1 (1.1)	7.2	5.2 (0.6)	7.4 (1.4)	6.6	4.2 (0.8)

[a] Unless otherwise indicated, data are expressed as mean (SD).
Abbreviations: HHS, Healthy Heart Score, BMI, body mass index (calculated as weight in Kg divided by height in m squared); JHS, Jackson Heart Study; HPFS, Health Professionals Follow-up Study; CARDIA, Coronary Artery Risk Development in Young Adults; NHS, Nurses' Health Study. [b]Standard deviations for mean HHS values were not available in published data from the HPFS and NHS.

Table S2: Model performance of Multivariate analysis including Lifestyle Factors.

**Incident Cardiovascular Events**	Women (n=1917) HR (95% CI)	Men (n=1124) HR (95% CI)	Total (n=3041) HR (95% CI)
Age, per 5 years	1.5 (1.37, 1.64)	1.47 (1.33, 1.64)	1.48 (1.38, 1.58)
Smoking			
Past	1.25 (0.76, 2.06)	0.99 (0.57, 1.72)	1.18 (0.82, 1.7)
Current	3.18 (1.85, 5.45)	2.01 (1.12, 3.58)	2.6 (1.76, 3.85)
Body mass index, kg/m^2	0.99 (0.97, 1.02)	1.01 (0.96, 1.05)	0.99 (0.97, 1.02)
Alcohol intake, per 10 g/day	1.07 (0.85, 1.34)	0.96 (0.82, 1.13)	1 (0.88, 1.13)
Physical activity, per 3 hours per week	0.995 (0.72, 1.38)	1.21 (0.87, 1.69)	1.1 (0.87, 1.39)
Diet score, per 1 point	0.98 (0.94, 1.03)	0.95 (0.9, 0.99)	0.97 (0.94, 0.995)
C-index (95% CI)	0.77 (0.72, 0.81)	0.75 (0.7, 0.81)	0.76 (0.73, 0.8)

Table S3. Model performance of the Healthy Heart Score in the Jackson Heart Study for Cardiovascular Events or death for the overall, in Participants with and without diabetes based on a) age only model; b) Healthy Heart Score

**Incident Cardiovascular Events**	Participants without Diabetes at Baseline (n=3007)	Participants with Diabetes at Baseline (n=707)
Hazard Ratio (95% CI) Age Alone	1.07 (1.06, 1.09)	1.04 (1.02, 1.06)
Hazard Ratio (95% CI) Healthy Heart Score	1.98 (1.76, 2.22)	1.56 (1.29, 1.89)
Harrell's Concordance Statistic (95% CI) - Age Alone	0.74 (0.71, 0.77)	0.62 (0.57, 0.67)
Harrell's Concordance Statistic – (95% CI) Healthy Heart Score	0.75 (0.71, 0.78)	0.62 (0.57, 0.67)
Time Dependent AUC (95% CI) – Age Alone		
1 year	0.76 (0.65, 0.88)	0.80 (0.5, 1)
3 years	0.76 (0.70, 0.83)	0.67 (0.45, 0.88)
5 years	0.76 (0.71, 0.82)	0.63 (0.45, 0.82)
Integrated Time-Dependent AUC – Age Alone	0.76	0.66
Time Dependent AUC (95% CI) – Healthy Heart Score		
1 year	0.78 (0.68, 0.88)	0.76 (0.46, 1.00)
3 years	0.77 (0.71, 0.82)	0.63 (0.41, 0.85)
5 years	0.77 (0.72, 0.82)	0.62 (0.41, 0.83)
Integrated Time-Dependent AUC– Healthy Heart Score	0.77	0.65

Figure S1. Formula to estimate the 20-Year Risk of CVD based on lifestyle predictors in women (Nurses' Health Study) and men (Health Professionals Follow-up)

WOMEN

20-year CVD risk (%) "Healthy Heart Score" = $[1 - 0.9660^{(\exp[W - 6.57301)}] \times 100\%$

where W= 0.10820 x age + 0.15285 (if **past smoker**) + 0.90138 (if **current smoker**) + 0.04676 × **BMI** – 0.01923 × **grams/d of alcohol** + 0.0004 × $(\text{grams/d of alcohol})^2$ – 0.029251 × **hours/week of physical activity** - 0.05113 × **diet score***

*Diet score = (0.03626 × grams/d of cereal fiber + 0.18283 [if fruits + vegetables ≥3 servings/d] + 0.14522 [if nuts 0.1-1 servings/d + 0.2444 [if nuts >1 servings/d]- 0.14631 × servings/d of sugar-sweetened beverages - 0.15624 × servings/d of red and processed meats)*10

MEN

20-year CVD risk (%) "Healthy Heart Score" = $[1 - 0.96368^{(\exp[W - 7.2437)}] \times 100\%$

where W= 0.13580 x age- 0.0005 x $(\text{age})^2$ + 0.06979 (if **past smoker**) + 0.42305 (if **current smoker**) +0.07424 × **BMI** – 0.00898 × **grams/d of alcohol** + 0.0001 × $(\text{grams/d of alcohol})^2$ – 0.01755 × **hours/week of physical activity** - 0.06691 × **diet score***

*Diet score = (0.01816× grams/d of cereal fiber + 0.08819 [if fruits + vegetables ≥3 servings/d] + 0.00535 [if nuts 0.1-1 servings/d + 0.14285 [if nuts >1 servings/d]- 0.14734 × servings/d of sugar-sweetened beverages - 0.07112 × servings/d of red and processed meats)*10
